# Inequities in the incidence and mortality due to COVID-19 in nursing homes in Barcelona by characteristics of the nursing homes

**DOI:** 10.1371/journal.pone.0269639

**Published:** 2022-06-13

**Authors:** Mayara Louise Torres, David Palma Díaz, Alba Oliver-Parra, Joan-Pau Millet, Delfí Cosialls, Montserrat Guillaumes, Cristina Rius, Hugo Vásquez-Vera

**Affiliations:** 1 Universitat Pompeu Fabra, Barcelona, Catalonia, Spain; 2 Universitat Autònoma de Barcelona, Barcelona, Catalonia, Spain; 3 Servei d’Epidemiologia, Agència de Salut Pùblica de Barcelona, Barcelona, Catalonia, Spain; 4 CIBER de Epidemiología y Salud Pública (CIBERESP), Madrid, Spain; 5 Consorci Sanitari de Barcelona, Barcelona, Catalonia, Spain; 6 Oficina de Residències de Barcelona, Barcelona, Catalonia, Spain; Max Stern Yezreel Valley College, ISRAEL

## Abstract

**Background:**

Residents of Nursing Homes (NHs) have suffered greater impacts from the COVID-19 pandemic. However, the rates of COVID-19 in these institutions are heterogeneously distributed. Describing and understanding the structural, functional, and socioeconomic differences between NHs is extremely important to avoid new outbreaks.

**Objectives:**

Analyze inequalities in the cumulative incidences (CIs) and in the mortality rates (MRs) due to COVID-19 in the NHs of Barcelona based on the characteristics of the NHs.

**Methods:**

Exploratory ecological study of 232 NHs. The dependent variables were the cumulative incidence and mortality rate due to COVID-19 in NHs between March and June 2020. Structural variables of the NHs were evaluated such as neighborhood socioeconomic position (SEP), isolation and sectorization capacity, occupancy, overcrowding and ownership.

**Results:**

The cumulative incidence and mortality rate were higher in the low SEP neighborhoods and lower in those of high SEP neighborhoods. Regarding the isolation and sectorization capacity, Type B NHs had a higher risk of becoming infected and dying, while Type C had a lower risk of dying than Type A. Greater overcrowding was associated with greater morbidity and mortality, and higher occupancy was associated with higher incidence. The risk of becoming infected and dying in public NHs was significantly higher than for-profit NH.

**Conclusions:**

The social components together with the functional and infrastructure characteristics of the NHs influence the cumulative incidence and the mortality rate by COVID-19. It is necessary to redefine the care model in the NHs to guarantee the health of the residents.

## Introduction

The progressive aging of the population is magnifying the health, social and economic consequences for today’s society. This demographic change is giving way to an increase in the elderly population, which implies a higher prevalence of chronic diseases and frailty, resulting in the loss of autonomy and assuming an increase in the demand for services such as nursing homes for the elderly (NHs) [1].

NHs present a series of characteristics that expose residents to an increased risk of respiratory diseases and outbreaks [2–6]. The conditions of these facilities and the susceptibility of their residents provide an environment conducive to the rapid spread of respiratory pathogens that can enter to the NHs by their staff, visitors and new or transferred residents; and once inside, spread rapidly among residents [3,7–9].

Before COVID-19 pandemic, influenza and other viruses such as parainfluenza, rhinovirus, adenovirus, metapneumovirus, other coronaviruses, and respiratory syncytial virus, have been described as outbreaks pathogens in NHs [10,11]. Previous studies show that NHs are not fully prepared for respiratory virus outbreaks, with some cases reporting that even routine infection control measures or vaccination were not enough to prevent illness, complications and deaths [12–16].

In 2020, elderly people, especially those living in NHs, were particularly affected by COVID-19. In an English study accounting of 9,000 NHs on the first wave of the pandemic, more than half of the NHs reported at least one confirmed case [17]. In the USA, in the same period, of 13,709 NHs, 39% reported at least one case [18]. Also, since the beginning of the pandemic, COVID-19 outbreaks in NHs have accounted for the high mortality worldwide [19–22]. By May, in Canada more than 80% of all COVID-19 deaths occurred in NHs [20], while in Europe, almost half of all deaths were occurring in NHs in several countries [21], where long-term care facilities, including NHs, registered 26 to 66% of all COVID-19 deaths [23].

In Catalonia there were more than 64,000 people living in NHs in 2020 with a mean age of 83 years old [24,25]. From the start of the pandemic until June 1, around 36,500 people living in NHs had been suspected of having COVID-19 and almost 14,000 had been confirmed, implying that approximately 80% of the elderly who lived in NHs could have suffered from the disease [25]. In terms of mortality, there were 3,965 deaths (confirmed and suspected cases) in the NHs, which accounted for 33% of all deaths in Catalonia due to this cause [25]. In Barcelona, since the beginning of the pandemic until the end of June, the highest cumulative incidence (CI) at 14 days per 100,000 inhabitants for the age group of 80–89 years old, was 902 in the general population (non-institutionalized elderly), while in the elderly living in NHs, it was 12,775, a CI more than 10 times higher [26]. Regarding mortality per 100,000 inhabitants, during the same period, the highest rate for the same age group was 200 in the general population, and 2,094 in residents of NHs, a value also more than 10 times higher [26]. As for older people, for the general population and for residents of 90 or more years old, the CI was 1,244 and 18,468, and the mortality rate was 701 and 5,279, respectively [26].

It has been shown that the risk of morbidity and mortality in NHs are heterogeneous according to the characteristics of the residents and the institutions [27–30]. Structural and functional characteristics of NHs, such as sharing common spaces, having frequent contacts with the same healthcare personnel, housing a large number of frail elderly people sharing a room, difficulties of sectorization and isolation and even the ownership of these institutions are determining factors that may increase the risk of infection by COVID-19 [2,3,7,8,20,31]. All this added to the axes of social inequality such as age, territory, or social class may explain that the distribution of the COVID-19 epidemic has been concentrated in this group with greater frequency and severity than in other population segments [32–34].

Given the high incidence and mortality rates due to COVID-19 in NHs in the city of Barcelona during the first wave of the current pandemic, it is essential to understand which characteristics of the NHs are related to a greater probability of being affected by COVID-19. Acknowledging the profile of the most affected NHs and their associated factors will make it possible to improve prevention and control measures in these centers. Thus, the objective of this study was to analyze differences in the cumulative incidence (CI) and mortality rate (MR) of COVID-19 between the NHs of the city of Barcelona, according to their socioeconomic, functional, and structural variables.

## Methods

### Design, study population and sources of information

**Fig 1:**
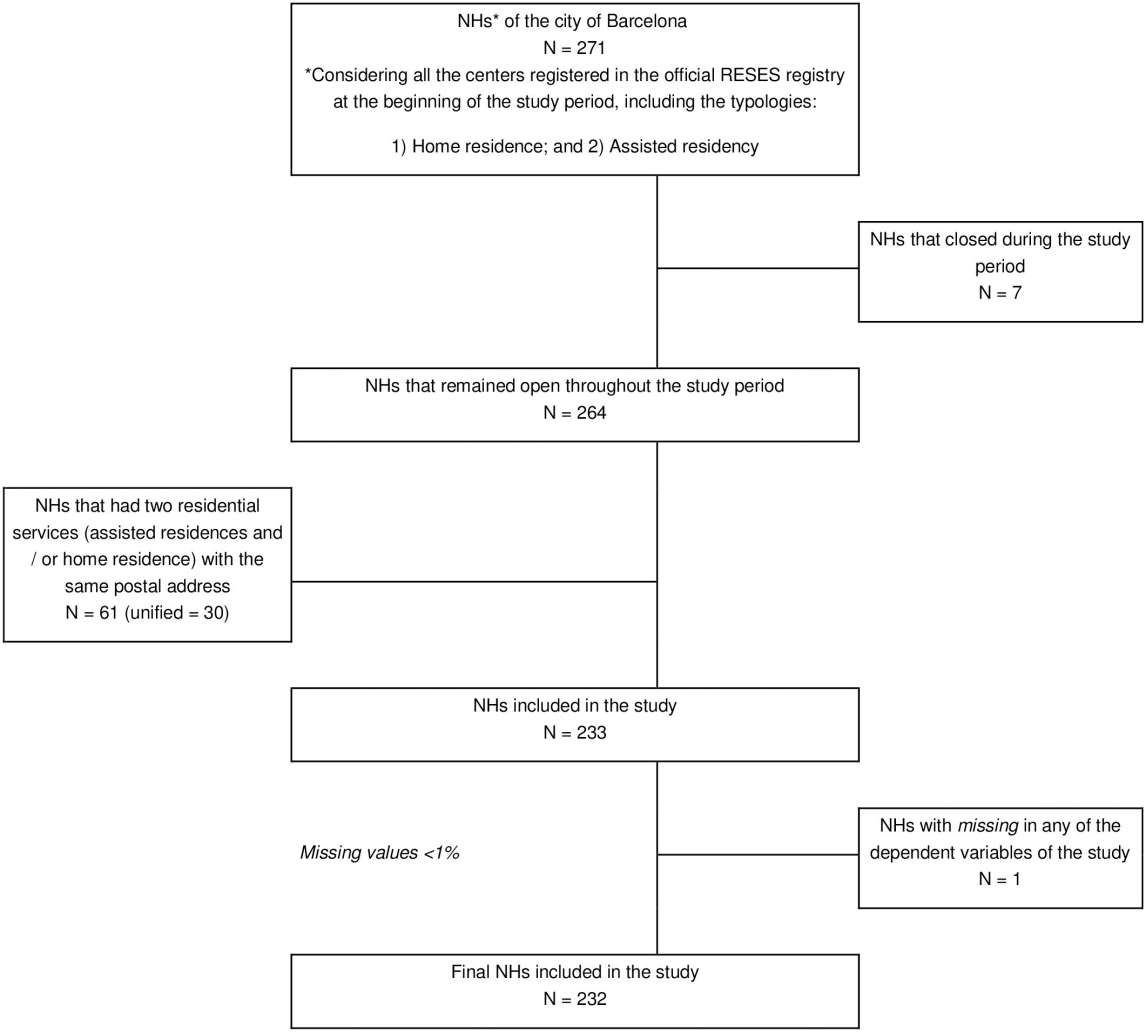
Flow diagram of the selection of the study population.

Ecological exploratory study of the NHs of the city of Barcelona in the period from March to June 2020. All the NHs of Barcelona that were cataloged in the RESES (Registry of Entities, Services and Social Establishments) as “assisted residence” or “home residence” were included in the study [35]. The NHs that closed during the study period were excluded, leaving an N of 232 residences (Fig 1).

The data on cases and deaths due to NHs were obtained from the GIR platform (Gestió d’Informació de les Residències), a tool developed during the pandemic by the Department of Social Rights of Catalonia (Departament de Drets Socials) to manage the pandemic in NHs. This platform, created using Microsoft Dynamics CRM (customer relationship management), contains information regarding the sociodemographic, functional, structural and health characteristics of the NHs and relies on administrative data and self-reported data from the NHs. Cases were defined from suspected and confirmed cases, while deaths were defined from deaths of residents identified as cases. These data were collated and corrected in case of underreporting with the records of the System of Information of the Service of Primary Care (SISAP—Sistema d’Informació dels Serveis d’Atenció Primària), which contain information from the primary care teams and funeral homes. The data on the structure and operation of the NHs were obtained from the GIR and the Internal Contingency Plans which is a document that stipulates action protocols for NHs and contains details of its structural data. The socioeconomic position data (SEP) of the NHs neighborhood were obtained from the Statistical Department of the Barcelona City Council [36].

The study complied with the declaration of Helsinki and adheres to the ethical regulations of the Barcelona´s Public Health Agency (Agència de Salut Pública de Barcelona). The data is available for researchers who meet the criteria for access to confidential data. We had access to the data as research staff of the Barcelona´s Health Consortium (Consorci Sanitari de Barcelona) and the Barcelona´s Public Health Agency, both as government institutions.

### Variables

The dependent variables of the study were the cumulative incidence (CI) and the mortality rate (MR) due to COVID-19 in NHs during the period March 1 to June 22, 2020. Both were defined as the number of incident cases or deaths during the study period divided by the number of residents at the beginning of the period per 100.

The explanatory variables were socioeconomic and structural indicators related to the NHs. In the first place, the social economic position (SEP) of the neighborhood where the residence is located was categorized into neighborhoods with upper middle income (> 126), medium (79–126) or low (<79), based on the Available Family Income Index, a compound indicator that allows knowing the relative SEP of each neighborhood of the city, where 100 corresponds to the mean value [36]. The isolation and sectorization capacity were categorized at the beginning of the pandemic according of: A: sufficient professionals and with the possibility of isolation and sectorization; B: sufficient professionals, without the capacity to sectorize by zones, but could isolate in individual rooms; C: without sufficient professionals and without the ability to isolate or sectorize [37]. The degree of occupancy of the center (ratio of the number of residents at the beginning of the period between the registration capacity) was categorized as "complete" or "partial", depending on whether the NHs had an occupancy greater or less than 95%, the maximum recommended occupancy by official regulations [38]. Crowding, measured by the ratio of residents at the beginning of the period by number of rooms, was categorized according to terciles into high (T3), medium (T2) and low crowding (T1). The ownership was categorized as NHs Private for-profit; Private not-for-profit; or Public [39].

### Analysis plan

A descriptive analysis was performed, presenting continuous and categorical variables as mean, median or absolute and percentage frequency (%), as appropriate. A bivariate analysis was performed between the quantitative dependent variables IA and TM, and the socioeconomic and structural variables of the NHs. For the categorical independent variables, the association between them was studied using the T-student test, ANOVA or Kruskal-Wallis, as appropriate. Finally, Poisson regression models were fitted to estimate the crude and adjusted relative risks (RRc and RRa, respectively) and their 95% confidence intervals (95% CI). The multivariate models to estimate the RRa included those variables that were significant in the crude models or that were considered relevant due to a previous bibliographic review.

These analyzes were conducted in Stata 13 statistical software.

### Ethics statement

To guarantee confidentiality of the data and records, we adhered to the regulations established by the Organic Law on the Protection of Personal Data 15/1999 in Spain, the European data protection Law 2018/1725 and to the ethical principles for human research defined by the Helsinki Declaration of 1964, revised and updated by the World Medical Organization (Edinburgh 2000). Data were collected for public health surveillance purposes. The information of this ecological study is aggregated data, does not identify or have personal information, and is free and available therefore the study did not required ethical approval.

## Results

**Fig 2:**
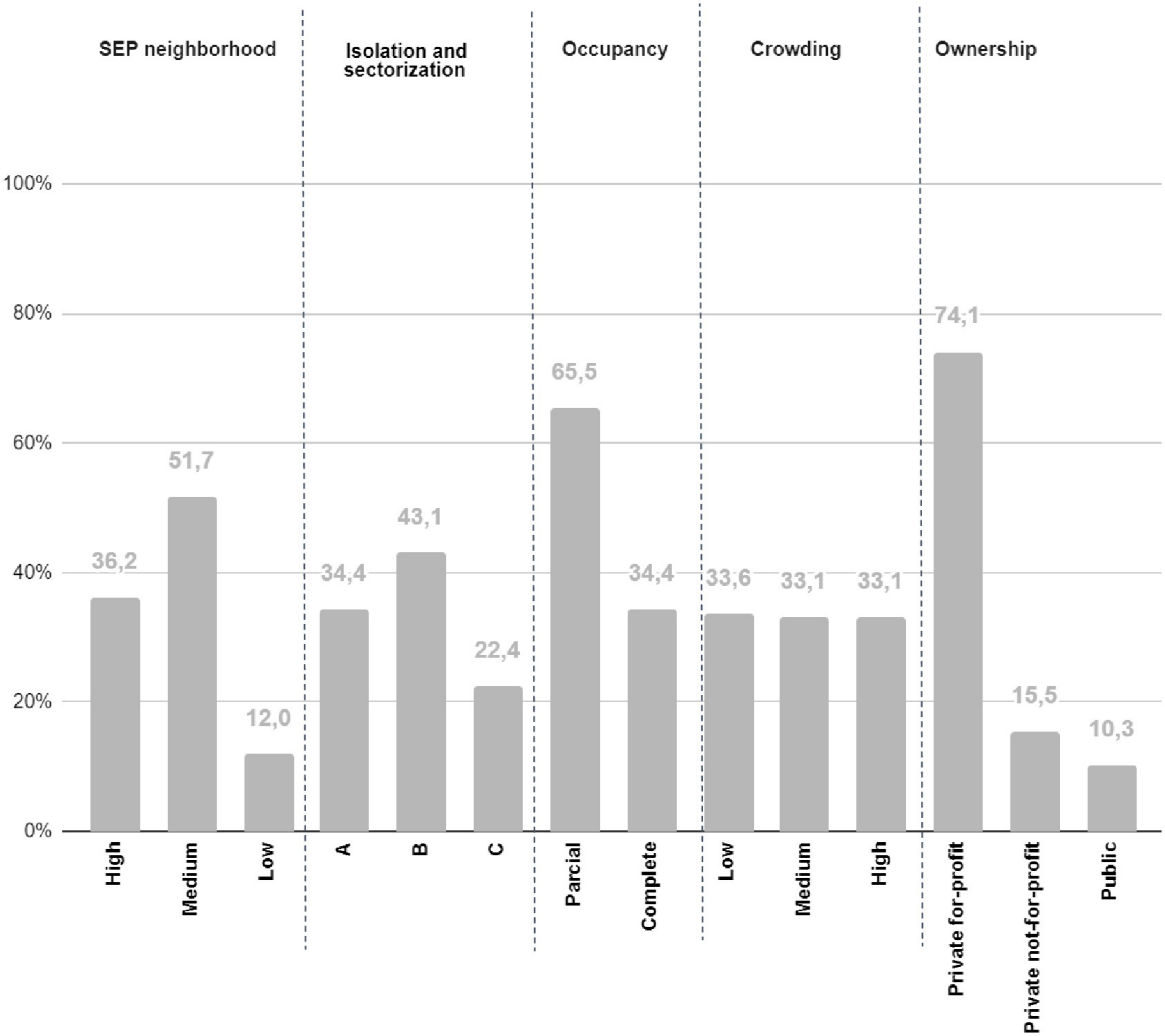
Characteristics of the NHs. Description of the characteristics of the NHs of the city of Barcelona in the period March-June 2020.

The mean CI was 36.7 x 100 residents, while the mean MR was 12.1 x 100 residents. Most of the NHs in Barcelona belong to neighborhoods with a medium income (51.7%), followed by neighborhoods with high income (36.3%) and low income (12%). Only 24.4% of the NHs have type A sectorization and isolation capacity, with the majority being type B (43.1%) and 22.4% of type C. 65.5% of the NHs had full occupancy and 33.1% had a high crowding. Most of the NHs are private for-profit owned (74.1%), 15.5% are private not-for-profit and only 10% are public owned (Fig 2).

### Cumulative incidence (CI) according to socioeconomic and structural variables of the NHs

Table 1 shows the cumulative incidence (CI) for COVID-19 during the period from March to June 2020 per 100 residents. The CI presents a greater magnitude in the NHs of neighborhoods with low SEP (44.2%), followed by neighborhoods with a medium (39.7%) and high SEP (29.8%). The risk of becoming infected by COVID-19 was 44% higher in the NHs of neighborhoods of low SEP (RRa: 1.44; 95% CI = 1.34–1.55) and 28% higher in the NHs of neighborhoods of medium SEP (RRa: 1.28; 95% CI = 1.21–1.34) compared with those in neighborhoods of high SEP.

**Table 1:** Cumulative incidence by COVID-19 of the nursing homes by socioeconomic and structural variables of the NHs in the period from March to June 2020 in Barcelona.

	BIVARIATE			CRUDE RR		ADJUSTED RR	
	Mean	Median	p value	Relative Risk (95% CI)	p value	Relative Risk (95% CI)	p value
**SEP of neighborhood**							
high	29.85	19.52	0.03 ^a^ *	1		1	
medium	39.75	39.84		1.33 (1.26–1.39)	<0.001*	1.28 (1.21–1.34)	<0.001*
low	44.21	50		1.48 (1.38–1.58)	<0.001*	1.44 (1.34–1.55)	<0.001*
**Isolation and sectorization capacity**							
A	33.27	27.77	0.81 ^b^	1		1	
B	40.30	39.41		1.21 (1.15–1.27)	<0.001*	1.25 (1.18–1.32)	<0.001*
C	28.70	17.64		0.86 (0.79–0.93)	<0.001*	0.93 (0.86–1.01)	0.10
**Occupancy**							
partial	32.94	22.55	0.19 ^a^	1		1	
complete	38.68	36		1.17 (1.12–1.22)	<0.001*	1.07 (1.02–1.12)	<0.001*
**Crowding**							
low	30.91	50.56	0.23 ^c^	1		1	
medium	41.40	70.31		1.33 (1.27–1.41)	<0.001*	1.43 (1.35–1.51)	<0.001*
high	37.87	71.87		1.22 (1.16–1.29)	<0.001*	1.36 (1.28–1.45)	<0.001*
**Ownership**							
private for-profit	36.63	34.54	0.35 ^b^	1		1	
private not-for-profit	32.05	20.02		0.87 (0.82–0.93)	<0.001*	1.02 (0.95–1.09)	0.46
public	44.19	46.14		1.20 (1.13–1.28)	<0.001*	1.15 (1.06–1.24)	<0.001*

SEP: Socioeconomic position; CI: Confidence Interval.

*P value <0.05;

^a^ Ttest;

^b^ ANOVA;

^c^ Kruskal-Wallis.

The CI was higher in NHs with type B isolation and sectorization capacity (40.3%) followed by type A (33.3%) and type C (28.70%). Regarding type A NHs, type B had a significantly higher risk (25%) of having cases (RRa: 1.25; 95% CI = 1.18–1.32).

Considering the occupancy, NHs of complete occupancy had a higher CI (38.6%) than those of partial occupancy (32.9%). The risk of becoming infected in complete occupancy NHs was 7% higher than in NHs with partial occupancy (RRa: 1.07; 95% CI = 1.02–1.12).

The CI was higher in NHs that presented medium crowding (41.4%), followed by high (37.8%) and low crowding (30.3%). Residents from NHs of medium crowding (RRa: 1.43; 95% CI = 1.35–1.51) and high crowding (RRa: 1.36; 95% CI = 1.28–1.45) had 43% and 36% greater risk of infection than in low crowded NHs.

Regarding the ownership, the CI was highest in the public owned NHs (44.1%), followed by the private for-profit (36.6%) and private not-for-profit (32.0%). Residents of public owned NHs had a 15% higher risk than those of private for-profit ownership (RRa: 1.15; 95% CI = 1.06–1.24), but the relationship with private not-for-profit NHs was not significant (RRa: 1, 02; 95% CI = 0.95–1.09).

### Mortality rate (MR) according to socioeconomic and structural variables of the NHs

Table 2 shows that the MR per 100 residents presented a higher magnitude in the neighborhoods with low SEP (15.9%), followed by those of medium (12.7%) and high (10.0%) SEP. The risk of dying from COVID-19 was 51% higher in the NHs of neighborhoods with low SEP (RRa: 1.51; 95% CI = 1.34–1.71), and 26% higher in the NHs of medium SEP (RRa: 1.26; 95% CI = 1.15–1.37), compared to those of neighborhoods with high SEP.

**Table 2:** Mortality rate of nursing homes according to socioeconomic and structural variables in the period from March to June 2020 in Barcelona city.

	BIVARIATE			CRUDE RR		ADJUSTED RR	
	Mean	Median	p value	Relative Risk (95% CI)	p value	Relative Risk (95% CI)	p value
**SEP of neighborhood**							
high	10.05	5.26	0.04 ^b^ *	1		1	
medium	12.70	10.50		1.26 (1.16–1.37)	<0.001*	1.26 (1.15–1.37)	<0.001*
low	15.98	11.25		1.58 (1.41–1.78)	<0.001*	1.51 (1.34–1.71)	<0.001*
**Isolation and sectorization capacity**							
A	11.95	7.69	0.07^b^	1		1	
B	13.21	11.40		1.10 (1.01–1.20)	0.02*	1.10 (1.00–1.21)	0.03*
C	8.26	4.65		0.69 (0.60–0.79)	<0.001*	0.67 (0.58–0.78)	<0.001*
**Occupancy**							
partial	11.63	5.88	0.63^a^	1		1	
complete	12.40	10.26		1.06 (0.98–1.15)	0.11	0.92 (0.85–1.00)	0.07
**Crowding**							
low	10.48	7.14	0.28^b^	1		1	
medium	12.70	10.93		1.21 (1.10–1.32)	<0.001*	1.34 (1.21–1.48)	<0.001*
high	13.24	8.69		1.26 (1.15–1.38)	<0.001*	1.49 (1.34–1.66)	<0.001*
**Ownership**							
private for-profit	11.84	7.92	0.33 ^b^	1		1	
private not-for-profit	11.35	7.22		0.95 (0.86–1.06)	0.43	1.19 (1.06–1.33)	<0.001*
public	15.38	15.52		1.29 (1.16–1.45)	<0.001*	1.30 (1.14–1.48)	<0.001*

SEP: Socioeconomic position; CI: Confidence Interval.

*P value <0.05;

^a^ Ttest;

^b^ ANOVA;

^c^ Kruskal-Wallis.

Regarding the capacity of isolation and sectorization, the MR was higher in type B NHs (13.2%) followed by type A (11.9%) and type C (8.2%). The risk of dying in type B NHs was 10% higher (RRa: 1.10; 95% CI = 1.00–1.21) and in type C it was 33% lower in relation to type A NHs (RRa: 0.67; 95% CI = 0.58–0.78).

Concerning the occupancy, the MR was higher in the NHs of complete occupancy (12.4%) than in those of partial occupancy (11.6%). In the adjusted analysis, complete occupancy was not associated with higher MR (RRa: 0.92; 95% CI = 0.85–1.00).

The MR was higher in NHs that present high crowding (13.2%), followed by those with medium (12.7%) and low crowding (10.48%). Residents with medium (RRa: 1.34; 95% CI = 1.21–1.48) and high crowding (RRa: 1.49; 95% CI = 1.34–1.61) had 34% and a 49% higher risk of dying than in low crowded NHs, respectively.

Relative to the ownerships, the MR was highest in the public NHs (15.3%), followed by private for-profit (11.8%) and the private not-for-profit (11.3%). Publicly owned NHs presented a 30% higher risk (RRa: 1.30; 95% CI = 1.14–1.48) and the private not-for-profit ones had a 19% higher risk (RRa: 1.19; 95% CI = 1.06–1.33) in relation to private for-profit NHs.

## Discussion

The results of this study suggest that the social components together with the functional and infrastructure characteristics of the NHs influence the incidence and mortality due to COVID-19. A socioeconomic gradient was observed in the distribution of CI and MR, with a higher frequency of both in the NHs of neighborhoods with low SEP. Regarding the isolation and sectorization capacity, type B NHs had a higher risk of having its residents becoming infected and dying, while type C had a lower risk of dying than type A. The data also associated a greater crowding with higher CI and MR and higher from COVID-19 were private for-profit NHs.

In the first place, the findings show that CI and MR during the first wave of COVID-19 in the NHs show a pattern depending on the neighborhood’s SEP, with the most disadvantaged areas being the most affected. These results are similar to official data that show territorial inequalities, supporting the evidence that social factors impact quality of life, and that these social determinants of health are worse in the more disadvantaged groups [32,40,41]. Territorial inequalities mean that people living in low-income neighborhoods do not have the same access to information, resources, services, and infrastructure as residents of high-income neighborhoods, which is reflected in their health status [19,32,42,43]. In S1 Table the characteristics of the NHs by SEP are observed.

The relationship between CI from COVID-19 and the crowding and occupancy of the NHs indicates that facilities with less crowding and occupancy have less incidence and mortality. Other studies also found similar results [30,44,45]. Social distancing, recommended as a form of contagious prevention, can be difficult in highly crowded NHs, where crowds are more common, healthcare personnel have contact with a greater number of residents, and there are greater difficulties in isolating residents positive, resulting in a greater probability of morbidity and mortality due to the virus [3,46].

Due to their poorer isolation and sectorization capacity, type B NHs present a greater risk than type A of both becoming infected and dying. On the contrary, it was observed that type C residences present better results than type A in mortality. In complementary analyses, it was found that the majority of type C NHs are medium SEP, low overcrowding, medium occupancy, and private for-profit ownership, while those of type A are mostly high SEP, have high occupancy, high overcrowding and private for-profit (S2 Table). When stratifying CI and MR by isolation and sectorization capacity and by SEP, we find that the risk of having cases in type B increases as the SEP decreases, when comparing to type A NHs (S3 and S4 Tables). Type B NHs have a 5% higher risk of having cases in high SEP (RR: 1.05; 95% CI = 0.98–1.05), 18% higher risk in medium SEP (RR: 1.18 95% CI = 1.09–1.27), and 36% higher risk in those with low SEP compared to type A NHs of the same SEP (RR: 1.36 95% CI = 1.19–1.55). In type C NHs, this relationship is not maintained since those with low SEP have a lower risk compared to type A (RR: 0.66 95% CI = 0.53–0.83). Regarding MR, in those of type B, the NHs of medium SEP present a greater risk compared to those of type A (RR: 1.41 95% CI = 1.23–1.63). While in the NHs of type C, those with low SEP are the ones that present better results in relation to type A (RR: 0.51 95% CI = 0.34–0.75). These differences according to strata make an interaction between the SEP and the isolation and sectorization capacity. Another explanation would be that the NHs were classified as A, B or C at the beginning of the pandemic, but their isolation and sectorization capacity, as well as the number of personnel, may have changed over the months, which may have had a significant impact on the ability of these institutions to adhere to standard infection control protocols [47,48]. It is also necessary to remember that type C NHs, due to their inability to isolate cases or sectorize the residence, received greater support from public organizations through the implementation of virus control measures such as the creation of contingency plans, the transfer of cases and contacts or the closure of some NHs [49,50]. In addition to the effect of support for type C residences, the lower MR suggests that the mechanisms that drive the risk of COVID-19 involvement in NHs are complex and may depend on the conjunction of other unmeasured factors.

Finally, the analyses show that publicly owned NHs had higher morbidity and mortality while private not-for-profit ones had higher mortality in relation to private for-profit NHs. These findings differ from those presented in other studies that include the ownership of the NHs [30,51,52]. It is presumed that an individual socioeconomic factor could have a participation on these results, due to a profile of residents with greater dependency and worse health status in the public and private not-for-profit NHs, since the priority of access to public services is determined by the degree of dependence and the economic capacity of the applicant, favoring the admittance of the most vulnerable people [53] and increasing inequality with residents of private for-profit NHs, who tend to present better health as they belong to a higher social class [32,54]. In addition, it is observed that of the private for-profit NHs, 39.5% are of high SEP, while, of the public ones, only 4.17% are of high SEP (S5 Table), which suggests that there may be other factors related to the SEP of the neighborhood that were not measured but could affect the health results of the people who reside in said territory. It is also important to mention that in Spain 44% of public NHs are managed by private companies or other entities [55].

This study has some limitations. First, the GIR database contains self-reported data, which may involve information biases. Unfortunately, some relevant variables that were on the GIR survey had to be excluded from the study as the information provided was not reliable, such as all the information about the number of caregivers and hours of work by profession, which consequently excluded the liability of rate of caregivers infected by COVID-19 during the period. It is important to consider that shortage of health personnel during the pandemic in the NHs was reported in various parts of the world. This shortage had a major impact not only on the ability of these institutions to adhere to standard infection control protocols, but also on their ability to provide necessary ongoing care that is not directly related to COVID-19 [47]. Second, this study uses data collected daily during the first months of the pandemic, which may be underestimated or overestimated due to difficulties encountered in this period to perform screening tests and due to possible differences in the validation of causes of death. Furthermore, the results may be biased by unmeasured interacting or confounding factors, such as the individual characteristics of the residents. Among its strengths, the sample includes all the NHs of Barcelona. To reduce the possibility of information biases, the GIR’s self-reported data were collated with data from the System of Information of the Service of Primary Care (SISAP), which contain information from the primary care teams and funeral homes. Regarding confusion and interaction biases, complementary analyzes were performed to better explain the results and can be seen in the supporting information area. The rate of visits was not measured in this study because in May of 2020 in Catalonia there were instructions from the responsible administrators to restrict visits at NHs to the maximum and admission was only allowed in cases of need or justified urgency during the period study [56,57]. The closure of residences to visitors represented one of the first measures adopted by these centers as an isolation mechanism against COVID-19. Differences on health services access were also not measured as all the population is covered by the publicly financed National Health System that provides universal coverage [58]. Considering all the limitations, this work offers results based on the most reliable existing data on the NHs during this period. With the increase in aging and life expectancy generating a greater number of residents in the NHs, more research is required to know the characteristics of these centers that are associated with a better quality of life for users and a lower risk of contracting communicable diseases. For this, it is essential to have better information systems that collect data on structural and organizational aspects of NHs and their residents, in order to build NHs basic services planning on evidence-based policies.

The way in which NHs were affected in the pandemic reveals structural and systemic problems, reinforcing the need to evolve towards a new model of care that is prepared to deal with new communicable diseases, promotes equity and protects the rights of residents. To achieve this change in model, social-health technical groups are necessary at different levels of the administration, to adjust the measures to be taken in times of outbreaks and redefine the basic service to guarantee the health and social well-being of the residents. Some recommendations derived from this study’s findings would include limiting the occupancy of the NHs and establishing the reservation of a number of rooms available for immediate use in case of need for isolation, establishing a maximum number of residents per room, increasing the investment of resources for public residences because they have a potentially more vulnerable resident population that needs greater care, and at the same time, rigorously regulate private NHs, avoiding the commercialization and dehumanization of their users.

## Supporting information

S1 Table Socioeconomic position of the nursing homes according to structural variables in the period from March to June 2020 in Barcelona city. (DOCX)

S2 Table Isolation capacity of the nursing homes and sectorization according to socioeconomic and structural variables in the period from March to June 2020 in Barcelona city. (DOCX)

S3 Table Cumulative incidence and socioeconomic position by structural variables of the nursing homes in the period from March to June 2020 in Barcelona city. (DOCX)

S4 Table Mortality rate and socioeconomic position by structural variables of the nursing homes in the period from March to June 2020 in Barcelona city. (DOCX)

S5 Table Ownership and financing of the nursing homes according to socioeconomic and structural variables in the period from March to June 2020 in Barcelona city. (DOCX)

S6 Table Crowding of the nursing homes according to socioeconomic and structural variables in the period from March to June 2020 in Barcelona city. (DOCX)

S7 Table Occupancy of the nursing homes according to socioeconomic and structural variables in the period from March to June 2020 in Barcelona city. (DOCX)

S1 Dataset (XLSX)
