# Correction: Inequities in the incidence and mortality due to COVID-19 in nursing homes in Barcelona by characteristics of the nursing homes

**DOI:** 10.1371/journal.pone.0315107

**Published:** 2024-12-02

**Authors:** The PLOS ONE Staff

The authors Alba Oliver-Parra, Joan-Pau Millet, Delfí Cosialls, Montserrat Guillaumes, Cristina Rius, Hugo Vásquez-Vera should not have been attributed equal contribution to this work. The second and third authors, David Palma Díaz and Alba Oliver-Parra, should be noted as contributing equally to this work.

The publisher apologizes for the error.
