# Supplementary material for: Inequities in the incidence and mortality due to COVID-19 in nursing homes in Barcelona by characteristics of the nursing homes
Source: PLoS One. 2022 Jun 13;17(6):e0269639. doi: 10.1371/journal.pone.0269639 (PMC9191699; doi:10.1371/journal.pone.0269639)
Supplement: S1 Table — (DOCX) [file pone.0269639.s001.docx]

|  | **High** | | | **Medium** | | | **Low** | | |  | |
| --- | --- | --- | --- | --- | --- | --- | --- | --- | --- | --- | --- |
|  | **CI** | **MR** | **Mean, Median or %*** | **CI** | **MR** | **Mean, Median or %*** | **CI** | **MR** | **Mean, Median or %*** | **Total** | **p value*** |
| **MR** | - | - | 10.05/5.26 | - | - | 12.70/10.50 | - | - | 15.98/15.07 | - | 0.04^a^ * |
| **CI** | - | - | 29.85/19.52 | - | - | 39.75/39.84 | - | - | 44.21/32.70 | - | 0.03^a^ * |
| **Isolation and sectorization capacity** |  |  |  |  |  |  |  |  |  |  |  |
| A | 29.80 | 12.40 | 34.52 | 35.87 | 10.14 | 20.00 | 38.06 | 15.74 | 28.57 | 26.29 | 0.15 ^b^ |
| B | 31.46 | 9.02 | 48.81 | 45.52 | 14.40 | 65.83 | 51.99 | 18.08 | 57.14 | 58.62 |  |
| C | 25.22 | 8.19 | 16.67 | 25.22 | 8.37 | 14.17 | 25.40 | 8.06 | 14.29 | 15.09 |  |
| total |  |  | 100.00 |  |  | 100.00 |  |  | 100.00 | 100.00 |  |
| **Occupancy** |  |  |  |  |  |  |  |  |  |  |  |
| partial | 27.71 | 9.06 | 38.10 | 36.35 | 12.79 | 30.00 | 36.65 | 15.03 | 42.86 | 34.48 | 0.29 ^b^ |
| complete | 31.17 | 10.66 | 61.90 | 41.20 | 12.65 | 70.00 | 49.88 | 16.69 | 57.14 | 65.52 |  |
| total |  |  | 100.00 |  |  | 100.00 |  |  | 100.00 | 100.00 |  |
| **Crowding** |  |  |  |  |  |  |  |  |  |  |  |
| low | 26.22 | 7.21 | 28.57 | 34.62 | 12.32 | 35.83 | 26.66 | 10.46 | 39.29 | 33.62 | 0.17 ^b^ |
| medium | 31.92 | 8.30 | 28.57 | 47.29 | 14.20 | 37.50 | 43.27 | 17.42 | 28.57 | 33.19 |  |
| high | 32.36 | 13.11 | 42.86 | 36.02 | 11.09 | 26.67 | 66.50 | 21.44 | 32.14 | 33.19 |  |
| total |  |  | 100.00 |  |  | 100.00 |  |  | 100.00 | 100.00 |  |
| **Ownership** |  |  |  |  |  |  |  |  |  |  |  |
| private for-profit | 31.41 | 9.90 | 80.95 | 38.57 | 12.36 | 74.17 | 48.80 | 17.61 | 53.57 | 74.14 | 0.00^b^ * |
| private not-for-profit | 23.51 | 11.16 | 17.86 | 37.36 | 10.75 | 14.17 | 41.48 | 14.64 | 14.29 | 15.52 |  |
| public | 19.04 | 3.57 | 1.19 | 50.12 | 17.21 | 11.67 | 37.77 | 13.85 | 32.14 | 10.34 |  |
| total |  |  | 100.00 |  |  | 100.00 |  |  | 100.00 | 100.00 |  |

CI: Cumulative Incidence, MR: Mortality Rate

**Values ​​are mean and median for continuous variables or % for categorical variables; * P value <0.05;

^a^ANOVA; ^b^Chi square.
