# Supplementary material for: Inequities in the incidence and mortality due to COVID-19 in nursing homes in Barcelona by characteristics of the nursing homes
Source: PLoS One. 2022 Jun 13;17(6):e0269639. doi: 10.1371/journal.pone.0269639 (PMC9191699; doi:10.1371/journal.pone.0269639)
Supplement: S2 Table — (DOCX) [file pone.0269639.s002.docx]

|  | **Isolation and sectorization capacity of the NHs** | | | | | | | | | | |
| --- | --- | --- | --- | --- | --- | --- | --- | --- | --- | --- | --- |
|  | **A** | | | **B** | | | **C** | | |  | |
|  | **CI** | **MR** | **Mean/ Median or %**** | **CI** | **MR** | **Mean/ Median or %**** | **CI** | **MR** | **Mean/ Median or %**** | **total** | **p value*** |
| **MR** |  |  | 11.95/7.69 |  |  | 13.21/11.40 |  |  | 8.26/4.65 | - | 0.07 ^a^ |
| **CI** |  |  | 33.27/27.77 |  |  | 40.30/39.41 |  |  | 28.70/17.64 | - | 0.03 ^a^* |
| **SEP** |  |  |  |  |  |  |  |  |  |  |  |
| high | 29.80 | 12.40 | 47.54 | 31.46 | 09.02 | 30.15 | 25.22 | 8.19 | 40.00 | 36.21 | 0.15 ^b^ |
| medium | 35.87 | 10.14 | 39.34 | 42.52 | 14.40 | 58.09 | 32.34 | 8.37 | 48.57 | 51.72 |  |
| low | 38.06 | 15.74 | 13.11 | 51.99 | 18.08 | 11.76 | 25.40 | 8.06 | 11.43 | 12.07 |  |
| total |  |  | 100.00 |  |  | 100.00 |  |  | 100.00 | 100.00 |  |
| **Occupancy** |  |  |  |  |  |  |  |  |  |  |  |
| partial | 34.35 | 14.08 | 21.31 | 38.03 | 13.28 | 33.82 | 20.92 | 6.52 | 60.00 | 10.34 | 0.00 ^b^* |
| complete | 32.98 | 11.87 | 78.69 | 41.46 | 13.18 | 66.18 | 40.38 | 10.87 | 40.00 | 39.66 |  |
| total |  |  | 100.00 |  |  | 100.00 |  |  | 100.00 | 100.00 |  |
| **Crowding** |  |  |  |  |  |  |  |  |  |  |  |
| low | 32.21 | 14.15 | 13.11 | 35.17 | 11.96 | 41.18 | 13.15 | 2.47 | 40.00 | 33.62 | 0.00 ^b^* |
| medium | 30.78 | 9.57 | 42.62 | 46.00 | 14.23 | 30.15 | 50.13 | 14.54 | 28.57 | 33.19 |  |
| high | 35.98 | 13.60 | 44.26 | 41.68 | 13.93 | 28.68 | 29.02 | 9.93 | 31.43 | 33.19 |  |
| total |  |  | 100.00 |  |  | 100.00 |  |  | 100.00 | 100.00 |  |
| **Ownership** |  |  |  |  |  |  |  |  |  |  |  |
| Private for-profit | 33.39 | 11.83 | 91.80 | 40.41 | 12.77 | 71.32 | 26.90 | 7.18 | 54.29 | 74.14 | <0.001 ^c^* |
| Private not-for-profit | 31.90 | 13.34 | 8.20 | 32.91 | 12.60 | 11.76 | 31.18 | 9.36 | 42.86 | 15.52 |  |
| public | - | - | 0.00 | 44.99 | 15.51 | 16.91 | 25.77 | 12.37 | 2.86 | 10.34 |  |
| total |  |  | 100.00 |  |  | 100.00 |  |  | 100.00 | 100.00 |  |

CI: Cumulative Incidence; MR: Mortality Rate; SEP: Socioeconomic Position.

**Values ​​are mean and median for continuous variables or % for categorical variables; * P value <0.05; ^a^ANOVA; ^b^Chi square; ^c^Fisher
