# Supplementary material for: Inequities in the incidence and mortality due to COVID-19 in nursing homes in Barcelona by characteristics of the nursing homes
Source: PLoS One. 2022 Jun 13;17(6):e0269639. doi: 10.1371/journal.pone.0269639 (PMC9191699; doi:10.1371/journal.pone.0269639)
Supplement: S3 Table — (DOCX) [file pone.0269639.s003.docx]

|  | **Cumulative incidence and socioeconomic position by structural variables of the NHs** | | | | | | | | | |
| --- | --- | --- | --- | --- | --- | --- | --- | --- | --- | --- |
|  | **Crude** | | **Adjusted by SEP** | | **Stratified by SEP** | | | | | |
|  |  |  |  |  | **High** | | **Medium** | | **Low** | |
|  | **RRc** | **95% CI** | **RRa** | **95% CI** | **RRs** | **95% CI** | **RRs** | **95% CI** | **RRs** | **95% CI** |
| **Ownership** |  |  |  |  |  |  |  |  |  |  |
| private for-profit | 1 |  | 1 |  | 1 |  | 1 |  | 1 |  |
| private not-for-profit | 0.87 | 0.82-0.93 | 0.87 | 0.82-0.93 | 0.74 | 0.66-0.83 | 0.96 | 0.89-1.05 | 0.85 | 0.71-1.00 |
| public | 1.20 | 1.13-1.28 | 1.07 | 1.00-1.15 | 0.60 | 0.38-0.95 | 1.29 | 1.19-1.40 | 0.77 | 0.68-0.88 |
| **Isolation and sectorization capacity** |  |  |  |  |  |  |  |  |  |  |
| A | 1 |  | 1 |  | 1 |  | 1 |  | 1 |  |
| B | 1.21 | 1.15-1.27 | 1.16 | 1.10-1.22 | 1.05 | 0.98-1.05 | 1.18 | 1.09-1.27 | 1.36 | 1.19-1.55 |
| C | 0.86 | 0.79-0.93 | 0.84 | 0.78-0.91 | 0.84 | 0.74-0.95 | 0.90 | 0.81-1.00 | 0.66 | 0.53-0.83 |
| **Crowding** |  |  |  |  |  |  |  |  |  |  |
| low | 1 |  | 1 |  | 1 |  | 1 |  | 1 |  |
| medium | 1.39 | 1.27-1.41 | 1.34 | 1.27-1.42 | 1.13 | 1.01-1.26 | 1.36 | 1.27-1.45 | 1.62 | 1.38-1.89 |
| high | 1.22 | 1.12-1.29 | 1.28 | 1.21-1.35 | 1.23 | 1.12-1.36 | 1.04 | 0.96-1.12 | 2.49 | 2.16-2.86 |
| **Occupancy** |  |  |  |  |  |  |  |  |  |  |
| partial | 1 |  | 1 |  | 1 |  | 1 |  | 1 |  |
| complete | 1.17 | 1.12-1.22 | 1.16 | 1.11-1.21 | 1.12 | 1.03-1.22 | 1.13 | 1.06-1.20 | 1.36 | 1.21-1.52 |

Adjusted and stratified analysis results from Poisson analysis.

SEP: socioeconomic position; RRc: Crude relative risk; RRa: Adjusted relative risk; RRs: Stratified relative risk; CI: Confidence Interval.
