# Supplementary material for: Inequities in the incidence and mortality due to COVID-19 in nursing homes in Barcelona by characteristics of the nursing homes
Source: PLoS One. 2022 Jun 13;17(6):e0269639. doi: 10.1371/journal.pone.0269639 (PMC9191699; doi:10.1371/journal.pone.0269639)
Supplement: S4 Table — (DOCX) [file pone.0269639.s004.docx]

| **Mortality rate and SEP by socioeconomic and structural variables of the Nursing Homes** | | | | | | | | | | |
| --- | --- | --- | --- | --- | --- | --- | --- | --- | --- | --- |
|  | **Crude** | | **Adjusted by SEP** | | **Stratified by SEP** | | | | | |
|  |  | |  | | **High** | | **Medium** | | **Low** | |
|  | **RRc** | **95% CI** | **RRa** | **95% CI** | **RRs** | **95% CI** | **RRs** | **95% CI** | **RRs** | **95% CI** |
| **Ownership** |  |  |  |  |  |  |  |  |  |  |
| Private for-profit | 1 |  | 1 |  | 1 |  | 1 |  | 1 |  |
| Private not-for-profit | 0.95 | 0.86-1.06 | 0.95 | 0.86-1.06 | 1.12 | 0.95-1.33 | 0.86 | 0.74-1.01 | 0.83 | 0.62-1.10 |
| public | 1.29 | 1.16-1.45 | 1.13 | 1.01-1.27 | 0.36 | 0.12-1.02 | 1.39 | 1.21-1.60 | 0.78 | 0.63-0.97 |
| **Isolation and sectorization capacity** |  |  |  |  |  |  |  |  |  |  |
| A | 1 |  | 1 |  | 1 |  | 1 |  | 1 |  |
| B | 1.10 | 1.01-1.20 | 1.07 | 0.98-1.16 | 0.72 | 0.62-0.84 | 1.41 | 1.23-1.63 | 1.14 | 0.93-1.41 |
| C | 0.69 | 0.60-0.79 | 0.68 | 0.59-0.78 | 0.66 | 0.53-0.81 | 0.82 | 0.67-1.01 | 0.51 | 0.34-0.75 |
| **Crowding** |  |  |  |  |  |  |  |  |  |  |
| low | 1 |  | 1 |  | 1 |  | 1 |  | 1 |  |
| medium | 1.21 | 1.10-1.32 | 1.22 | 1.11-1.34 | 1.15 | 0.93-1.41 | 1.15 | 1.02-1.29 | 1.66 | 1.30-2.13 |
| high | 1.26 | 1.15-1.38 | 1.32 | 1.20-1.45 | 1.81 | 1.52-2.16 | 0.90 | 0.78-1.02 | 1.04 | 1.62-2.58 |
| **Occupancy** |  |  |  |  |  |  |  |  |  |  |
| partial | 1 |  | 1 |  | 1 |  | 1 |  | 1 |  |
| complete | 1.06 | 0.98-1.15 | 1.06 | 0.98-1.15 | 1.17 | 1.02-1.35 | 0.98 | 0.88-1.10 | 1.11 | 0.91-1.34 |

Adjusted and stratified analysis results from Poisson analysis.

SEP: socioeconomic position; RRc: Crude relative risk; RRa: Adjusted relative risk; RRs: Stratified relative risk; CI: Confidence Interval.
