# Supplementary material for: Inequities in the incidence and mortality due to COVID-19 in nursing homes in Barcelona by characteristics of the nursing homes
Source: PLoS One. 2022 Jun 13;17(6):e0269639. doi: 10.1371/journal.pone.0269639 (PMC9191699; doi:10.1371/journal.pone.0269639)
Supplement: S5 Table — (DOCX) [file pone.0269639.s005.docx]

|  | **Ownership and Financing of the Nursing Homes** | | | | | | | | | | |
| --- | --- | --- | --- | --- | --- | --- | --- | --- | --- | --- | --- |
|  | **Private for-profit** | | | **Private not-for-profit** | | | **Public** | | |  | |
|  | **CI** | **MR** | **Mean/ Median or %**** | **CI** | **MR** | **Mean/ Median or %**** | **CI** | **MR** | **Mean/ Median or %**** | **Total** | **p value** |
| **MR** | **-** | - | 11.84/7.92 | - | - | 11.35/7.22 | - | - | 15.38/15.52 | - | 0.33 ^b^ |
| **CI** | **-** | - | 36.63/34.54 | - | - | 32.05/20.02 | - | - | 44.19/46.14 | - | 0.35 ^b^ |
| **SEP** |  |  |  |  |  |  |  |  |  |  |  |
| high | 31.41 | 9.90 | 39.53 | 23.51 | 11.16 | 41.67 | 19.04 | 3.57 | 4.17 | 36.21 | <0.001 ^a^* |
| medium | 38.57 | 12.36 | 51.74 | 37.36 | 10.75 | 47.22 | 50.12 | 17.21 | 58.33 | 51.72 |  |
| low | 48.80 | 17.61 | 8.72 | 41.48 | 14.64 | 11.11 | 37.77 | 13.85 | 37.50 | 12.07 |  |
| total |  |  | 100.00 |  |  | 100.00 |  |  | 100.00 | 100.00 |  |
| **Isolation and sectorization capacity** |  |  |  |  |  |  |  |  |  |  |  |
| A | 33.39 | 11.83 | 32.56 | 31.90 | 13.34 | 13.89 | - | - | 0.00 | 26.29 | <0.001 ^c^* |
| B | 40.41 | 12.77 | 56.40 | 32.91 | 12.60 | 44.44 | 44.99 | 15.51 | 95.83 | 58.62 |  |
| C | 26.90 | 07.18 | 11.05 | 31.18 | 9.36 | 41.67 | 25.77 | 12.37 | 4.17 | 15.09 |  |
| total |  |  | 100.00 |  |  | 100.00 |  |  | 100.00 | 100.00 |  |
| **Occupancy** |  |  |  |  |  |  |  |  |  |  |  |
| partial | 33.84 | 11.97 | 33.14 | 26.03 | 9.45 | 47.22 | 43.96 | 14.66 | 25.00 | 10.34 | 0.25 ^a^ |
| complete | 38.01 | 11.78 | 66.86 | 37.44 | 13.06 | 52.78 | 44.27 | 15.62 | 75.00 | 39.66 |  |
| total |  |  | 100.00 |  |  | 100.00 |  |  | 100.00 | 100.00 |  |
| **Crowding** |  |  |  |  |  |  |  |  |  |  |  |
| low | 32.24 | 10.78 | 23.84 | 19.59 | 5.85 | 52.78 | 39.84 | 14.69 | 75.00 | 33.62 | <0.001 ^a^* |
| medium | 39.96 | 12.32 | 37.21 | 45.30 | 13.57 | 22.22 | 53.57 | 16.09 | 20.83 | 33.19 |  |
| high | 36.14 | 12.04 | 38.95 | 46.57 | 20.99 | 25.00 | 75.60 | 24.39 | 4.17 | 33.19 |  |
| total |  |  | 100.00 |  |  | 100.00 |  |  | 100.00 | 100.00 |  |

CI: Cumulative Incidence; MR: Mortality Rate; SEP: Socioeconomic Position.

**Values ​​are mean and median for continuous variables or % for categorical variables; * P value <0.05; ^a^Chi square; ^b^ANOVA; ^c^Fisher.
