# Supplementary material for: Inequities in the incidence and mortality due to COVID-19 in nursing homes in Barcelona by characteristics of the nursing homes
Source: PLoS One. 2022 Jun 13;17(6):e0269639. doi: 10.1371/journal.pone.0269639 (PMC9191699; doi:10.1371/journal.pone.0269639)
Supplement: S6 Table — (DOCX) [file pone.0269639.s006.docx]

|  | **Nursing Homes crowding** | | | | | | | | | | |
| --- | --- | --- | --- | --- | --- | --- | --- | --- | --- | --- | --- |
|  | **Low** | | | **Medium** | | | **High** | | |  |  |
|  | **CI** | **MR** | **Mean/ Median or %**** | **CI** | **MR** | **Mean/ Median or %**** | **CI** | **MR** | **Mean/ Median or %**** | **total** | **p value** |
| **CI** | - | - | 30.91/ | - | - | 41.40 | - | - | 37.87 | - | 0.23 ^d^ |
| **MR** | - | - | 10.48/7.14 | - | - | 12.70/10.93 | - | - | 13.24/8.69 | - | 0.21 ^c^ |
| **SEP** |  |  |  |  |  |  |  |  |  |  |  |
| high | 26.22 | 7.21 | 30.77 | 29.71 | 8.30 | 31.17 | 32.36 | 13.11 | 46.75 | 36.21 | 0.17 ^a^ |
| medium | 34.62 | 12.32 | 55.13 | 47.29 | 14.20 | 58.44 | 36.02 | 11.09 | 41.56 | 51.72 |  |
| low | 26.66 | 10.46 | 14.10 | 43.27 | 17.42 | 10.39 | 66.50 | 21.44 | 11.69 | 12.07 |  |
| total |  |  | 100.00 |  |  | 100.00 |  |  | 100.00 | 100.00 |  |
| **Isolation and sectorization capacity** |  |  |  |  |  |  |  |  |  |  |  |
| A | 32.21 | 14.15 | 10.26 | 30.78 | 9.57 | 33.77 | 35.98 | 13.60 | 35.06 | 26.29 | 0.00 ^a^* |
| B | 35.17 | 11.96 | 71.79 | 46.00 | 14.23 | 53.25 | 41.68 | 13.93 | 50.65 | 58.62 |  |
| C | 13.15 | 2.47 | 17.95 | 50.13 | 14.54 | 12.99 | 29.02 | 9.93 | 14.29 | 15.09 |  |
| total |  |  | 100.00 |  |  | 100.00 |  |  | 100.00 | 100.00 |  |
| **Occupancy** |  |  |  |  |  |  |  |  |  |  |  |
| partial | 27.93 | 9.86 | 50.00 | 38.40 | 12.30 | 35.06 | 36.38 | 15.28 | 18.18 | 34.48 | <0.001 ^a^* |
| complete | 33.90 | 11.10 | 50.00 | 43.02 | 12.91 | 64.94 | 38.20 | 12.79 | 81.82 | 65.52 |  |
| total |  |  | 100.00 |  |  | 100.00 |  |  | 100.00 | 100.00 |  |
| **Ownership** |  |  |  |  |  |  |  |  |  |  |  |
| Private for-profit | 32.24 | 10.78 | 52.56 | 39.96 | 12.32 | 83.12 | 36.14 | 12.04 | 87.01 | 74.14 | <0.001 ^b^* |
| Private not-for-profit | 19.59 | 5.85 | 24.36 | 45.30 | 13.57 | 10.39 | 46.57 | 20.99 | 11.69 | 15.52 |  |
| public | 39.84 | 14.69 | 23.08 | 53.57 | 16.09 | 6.49 | 75.60 | 24.39 | 1.30 | 10.34 |  |
| total |  |  | 100.00 |  |  | 100.00 |  |  | 100.00 | 100.00 |  |

CI: Cumulative Incidence; MR: Mortality Rate; SEP: Socioeconomic Position.

**Values ​​are mean and median for continuous variables or % for categorical variables; *P value <0.05; ^a^Chi Square; ^b^Fisher; ^c^ANOVA; ^d^Kruskall-Wallis.
