# Supplementary material for: Inequities in the incidence and mortality due to COVID-19 in nursing homes in Barcelona by characteristics of the nursing homes
Source: PLoS One. 2022 Jun 13;17(6):e0269639. doi: 10.1371/journal.pone.0269639 (PMC9191699; doi:10.1371/journal.pone.0269639)
Supplement: S7 Table — (DOCX) [file pone.0269639.s007.docx]

|  | **Nursing Homes occupacy** | | | | | | | |
| --- | --- | --- | --- | --- | --- | --- | --- | --- |
|  | **Partial** | | | **Complete** | | |  |  |
|  | **CI** | **MR** | **Mean/ Median or %**** | **CI** | **MR** | **Mean/ Median or %**** | **total** | **p value** |
| **CI** | - | - | 32.94/22.55 | - | - | 38.68/36.00 | - | 0.19 ^b^ |
| **MR** | - | - | 11.63/5.88 | - | - | 12.40/10.26 | - | 0.63 ^b^ |
| **SEP** |  |  |  |  |  |  |  |  |
| high | 27.71 | 09.06 | 40.00 | 31.17 | 10.66 | 34.21 | 36.21 | 0.29 ^a^ |
| medium | 36.35 | 12.79 | 45.00 | 41.20 | 12.65 | 55.26 | 51.72 |  |
| low | 36.65 | 15.03 | 15.00 | 49.88 | 16.69 | 10.53 | 12.07 |  |
| total |  |  | 100.00 |  |  | 100.00 | 100.00 |  |
| **Isolation and sectorization capacity** |  |  |  |  |  |  |  |  |
| A | 34.35 | 14.08 | 16.25 | 32.98 | 11.38 | 31.58 | 26.29 | <0.001 ^a^* |
| B | 38.03 | 13.28 | 57.50 | 41.46 | 13.18 | 59.21 | 58.62 |  |
| C | 20.92 | 6.52 | 26.25 | 40.38 | 10.87 | 9.21 | 15.09 |  |
| total |  |  | 100.00 |  |  | 100.00 | 100.00 |  |
| **Crowding** |  |  |  |  |  |  |  |  |
| low | 27.93 | 9.86 | 48.75 | 33.90 | 11.10 | 25.66 | 33.62 | <0.001 ^a^* |
| medium | 38.40 | 12.30 | 33.75 | 43.02 | 12.91 | 32.89 | 33.19 |  |
| high | 36.38 | 15.28 | 17.50 | 38.20 | 12.79 | 41.45 | 33.19 |  |
| total |  |  | 100.00 |  |  | 100.00 | 100.00 |  |
| **Ownership** |  |  |  |  |  |  |  |  |
| Private for-profit | 33.84 | 11.97 | 71.25 | 38.01 | 11.78 | 75.66 | 74.14 | 0.15 ^a^ |
| Private not-for-profit | 26.03 | 9.45 | 21.25 | 37.44 | 13.06 | 12.50 | 15.52 |  |
| public | 43.96 | 14.66 | 7.50 | 44.27 | 15.62 | 11.84 | 10.34 |  |
| total |  |  | 100.00 |  |  | 100.00 | 100.00 |  |

CI: Cumulative Incidence; MR: Mortality Rate; SEP: Socioeconomic Position.

**Values ​​are mean and median for continuous variables or % for categorical variables;* P value <0.05; ^a^Chi Square; ^b^ANOVA.
